# In-hospital mortality and SpO2 incritical care patients with cerebral injury: data from the MIMIC‑IV Database

**DOI:** 10.1186/s12871-022-01933-w

**Published:** 2022-12-12

**Authors:** Haoyang Yin, Rui Yang, Yun Xin, Tao Jiang, Dong Zhong

**Affiliations:** Department of Neurosurgery, The First Affiliated Hospital of Chongqing Medical University, Chongqing, China

**Keywords:** Blood oxygen saturation, Oxygen therapy, Craniocerebral disease, Subarachnoid hemorrhage, Intensive care unit

## Abstract

**Background:**

Evidence regarding the relationship between in-hospital mortality and SpO2 was low oxygen saturations are often thought to be harmful, new research in patients with brain damage has found that high oxygen saturation actually enhances mortality. However, there is currently no clear study to point out the appropriate range for oxygen saturation in patients with craniocerebral diseases.

**Methods:**

By screening all patients in the MIMIC IV database, 3823 patients with craniocerebral diseases (according to ICD-9 codes and ICD-10) were selected, and non-linear regression was used to analyze the relationship between in-hospital mortality and oxygen saturation. Covariates for all patients included age, weight, diagnosis, duration of ICU stay, duration of oxygen therapy, etc.

**Results:**

In-hospital mortality in patients with TBI and SAH was kept to a minimum when oxygen saturation was in the 94–96 range. And in all patients, the relationship between oxygen saturation and in-hospital mortality was U-shaped. Subgroup analysis of the relationship between oxygen saturation and mortality in patients with metabolic encephalopathy and other encephalopathy also draws similar conclusions In-hospital mortality and oxygen saturation were all U-shaped in patients with subarachnoid hemorrhage, metabolic and toxic encephalopathy, cerebral infarction, and other encephalopathy, but the nonlinear regression was statistically significant only in patients with cerebral infarction (*p* for nonlinearity = 0.002).

**Conclusion:**

Focusing too much on the lower limit of oxygen saturation and ignoring too high oxygen saturation can also lead to increase in-hospital mortality. For patients with TBI and SAH, maintaining oxygen saturation at 94–96% will minimize the in-hospital mortality of patients.

## Introduction

Neurological diseases are currently the major cause of disability worldwide, and the prevalence of neurodegenerative disorders is rising as the population ages [1]. Encephalopathy is a condition in which the degree or content of consciousness is disrupted owing to brain malfunction, which can be caused by global or localized brain injury [2]. Cerebral injury has received extensive focus owing to its higher incidence and fatality rate [3]. Cerebral infarction, spontaneous subarachnoid hemorrhage, metabolic or toxic encephalopathy, traumatic brain injury, and other craniocerebral disorders are all common [4]. After cardiovascular illnesses and malignancies, neurological disorders ranked third in the EU, accounting for 19.5% (18.0–21.3) of total fatalities [5].

Adequate tissue oxygenation, which is dependent on blood oxygenation, is required for patients with craniocerebral disorders life. A large amount of oxygen is often supplied in the intensive care unit (ICU) in order to treat or prevent hypoxia. Recent evidence suggests that high blood oxygenation may produce vasoconstriction in critical system, such as the cerebral or coronary, as well as the production of free radicals, which can harm cells [6]. Direct lung toxicity and atelectasis may arise as a result of high inspired oxygen concentrations driving excessive blood oxygenation [7]. Oxygen saturation and partial pressure of oxygen are commonly used indicators to monitor the level of blood oxygen in patients. Compared with the partial pressure of oxygen obtained from the blood gas analysis, the blood oxygen saturation obtained from the peripheral pulse oximetry is easier to monitor, observe and non-invasive. For a long time, oxygen was thought to have consistently positive benefits [8]. However, an observational research using data from large ICU Databases discovered a U-shaped connection between SpO2 and mortality in the study participants [9]. In many cases, there are no definite oxygen treatment criteria that are followed. Yet, the best SpO2 range for a better clinical result in critical care patients with encephalopathy is unknown.

The central thesis of this paper is that using a vast database to determine the ideal SpO2 range linked with a survival benefit in patients with craniocerebral disorders. In patients with craniocerebral disorders, we postulate that there is a nonlinear relationship between SpO2 and in-hospital mortality.

## Materials and methods

### Data description

The present study utilizes the fourth edition of the Medical Information Mart for Intensive Care database (MIMIC-IV, v1.0) to analyses the relationship between in-hospital mortality and SpO2 in critical care patients with encephalopathy. The MIMIC-IV [10] database is a massive, open-access single-center critical care database that includes patients hospitalized to the Beth Israel Deaconess Medical Center in Boston from 2008 to 2019. Patients with Traumatic Brain Injury (TBI), metabolic/toxic encephalopathy, Subarachnoid Hemorrhage (SAH), other Intracerebral Hemorrhage (ICH), cerebral infarction, and other encephalopathies were discovered by International Classification of Disease, the Ninth Version and Tenth Version.

Firstly, patients above the age of 90 were filtered out. Patients who had been in the ICU for less than 48 h and had fewer than 24 SpO2 measures were eliminated. Then, SpO2 while on oxygen treatment was the key independent variable. The primary independent variable was SpO2 while on oxygen therapy, where oxygen therapy can be supplemental oxygen such as a nasal cannula, non-invasive, and invasive ventilation. We took the median of the SpO2 measurements during oxygen therapy as a measure of the central tendency of oxygen exposure. As a measure of the central tendency of oxygen exposure, we used the median of SpO2 readings taken during oxygen treatment. The Strengthening the Reporting of Observational Studies in Epidemiology (STROBE) statement was used to report this research [11]. All of the participants, we identified 403 patients with ICH, 1236 patients with metabolic or toxic encephalopathy, 431 patients with SAH,39 patients with TBI, 936 patients with cerebral infarction, and 778 patients with other encephalopathies.

### Variables

On the first day of ICU admission, the following variables were extracted from the MIMIC-IV databases: age, sex, weight, first care unit, Sequential Organ Failure Assessment (SOFA) score, Acute Physiology and Chronic Health Evaluation-III(APACHE-III), Glasgow Coma Scale (GCS), mechanical ventilation, comorbidities. Within twenty-four hours after ICU admission, SOFA score, APACHE, and GCS were determined. Comorbidities, including congestive heart failure, chronic pulmonary diseases, renal diseases were identified by the Ninth and Tenth Versions of the International Classification of Disease. Ventilation type, including supplemental oxygen, invasive ventilation, tracheostomy and high flow. The outcome was hospital mortality.

### Statistical analysis

The fact that both hypoxemia and hyperoxemia are linked with unfavorable outcomes shows that the connection between SpO2 and mortality is nonlinear. We utilized Generalized additive models to assess the relationship between median SpO2 and mortality while adjusting for age, weight, gender, SOFA score on the first day of the ICU stay, and oxygen treatment duration. The research participants were divided into two groups: survivors and nonsurvivors. Because the data was not normally distributed, the median and interquartile range (IQR) for continuous variables were used. The findings were reported as numbers and percentages for categorical factors. Continuous and categorical variables were analyzed using Mann–Whitney U-tests and χ2 tests, respectively.

SpO2 measures a patient's blood oxygen saturation and also determines whether or not the patient is Hypoxemic [12]. Therefore, we divided the patient's SpO2 into five grades (< 92, 92–94, 94–96, 96–98, > 98) to compare patients with different levels of oxygen saturation. The hazard ratios and 95 percent confidence intervals (CIs) of the link between SpO2 and in-hospital mortality were calculated using Cox regression analysis. Considering that the presence of hypoxemia in a small number of patients affected the overall prediction results, appropriate adjustments were made in the spline model.

To test the robustness of our results, we utilized two models for Cox regression analysis. Model 1 investigated the link between SpO2 and mortality without taking into account any variables. Model 2 was created using a forward stepwise regression strategy to identify baseline variables that were clinically relevant or had a univariate mortality connection. As a result, characteristics such GCS score, SOFA score, admission type, martial statue, mechanical ventilation usage, and comorbidities were eventually added.

The statistical analyses were performed using STATA software (version 16.0). All statistical tests were two-sided, and *p* < 0.05 was considered statistically significant.

## Results

**Fig. 1:**
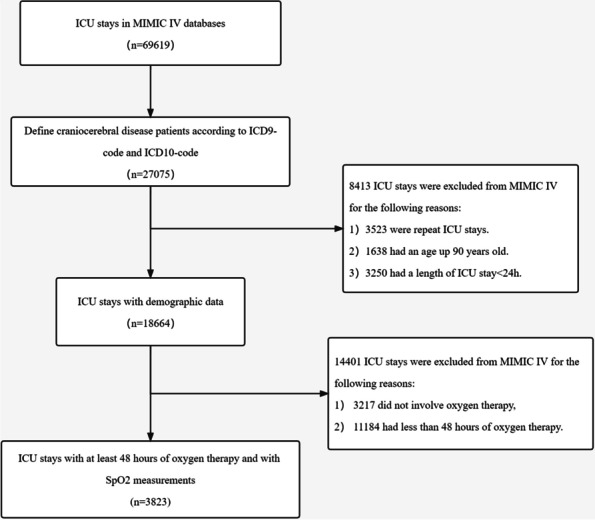
Patient selection flowchart. ICD10, tenth version of the International Classification of Disease, ICD9, ninth version of the International Classification of Disease, ICU, intensive care unit, MIMIC-IV, Medical Information Mart for Intensive Care fourth edition, SpO2, pulse oximetry-derived oxygen saturation

In total, 3823 patients out of a total of 69,619 patients in the MIMIC IV database were screened to fulfill the requirements by the application of inclusion and exclusion criteria (Fig. 1).

Table 1 summarizes the demographic and clinical characteristics. There were 1651(43.4%) women in total among the participants. The mean age of all participants was 68 [IQR 57–77] years, and the median ­SpO2 was 97%. Among them, 1022 (26.7%) patients died during hospitalization. Patients in the non-survival group were generally older and more critically ill than those in the survival group (age 69 [IQR 58–78] years vs. 67 [IQR 56–76] years, *p* < 0.001; SOFA score 9 [IQR 6–12] vs. 7 [IQR 4–10], *p* < 0.001; APACHE III 80 [IQR 61–101] vs. 59 [IQR 44–80], *p* < 0.001; GCS 7 [IQR 3–12] vs. 10 [IQR 7–13], *p* < 0.001). The study showed that the patients in the non-survival group were lighter (weight 78.9 [IQR 66.1–94.5] kg vs. 81.3 [IQR 68.0–98.0] kg, *p* < 0.001), but taking into account height and other factors, it was not meaningful. Patients in the non-surviving group were more likely to have kidney disease than those in the surviving group. Contrary to what we usually think, the oxygen saturation in the non-surviving group was higher than that in the survival group. There was no significant difference in the length of stay in the ICU between the two groups (8.2 [IQR 4.9–14.6] d vs. 8.1 [IQR 4.7–13.6], *p* = 0.136). Patients with different ventilation status also showed different survival rates, with higher mortality in patients using invasive ventilation.

**Table 1:** Baseline characteristics of all participants according to survival status

Characteristic	All (*n* = 3823)	Survival (*n* = 2801)	Non-survival (*n* = 1022)	*P* value
Female sex, n (%)	1651 (43.4)	1208 (43.4)	443 (43.5)	0.96
Age, Median (IQR) (year)	68 (57, 77)	67 (56, 76)	69 (58, 78)	< 0.001
Weight, Median (IQR) (kg)	80.5 (68.0, 97.1)	81.3 (68.5, 98.0)	78.9 (66.1, 94.5)	< 0.001
Diagnose, n (%)				
ICH (non-SAH)	403 (10.5)	257 (9.2)	146 (14.3)	
Metabolic/toxic encephalopathy	1236 (32.3)	934 (33.3)	302 (29.5)	
SAH	431 (11.3)	328 (11.7)	103 (10.1)	
TBI	39 ( 1.0)	33 (1.2)	6 (0.6)	
Cerebral infarction	936 (24.5)	723 (25.8)	213 (20.8)	
Other encephalopathies	778 (20.4)	526 (18.8)	252 (24.7)	
Severity scores, median (IQR)				
APACHE III	65.0 (47.0, 86.0)	59.0 (44.0, 80.0)	80.0 (61.0, 101.0)	< 0.001
GCS	9.0 (6.0, 13.0)	10.0 (7.0, 13.0)	7.0 (3.0, 12.0)	< 0.001
SOFA	8.0 (5.0, 11.0)	7.0 (4.0, 10.0)	9.0 (6.0, 12.0)	< 0.001
Comorbidities, n (%)				
Congestive heart failure	1019 (26.8)	716 (25.7)	303 (29.8)	0.014
Chronic pulmonary disease	1097 (28.8)	825 (29.6)	272 (26.7)	0.088
Renal disease	221 ( 5.8)	127 (4.6)	94 (9.2)	< 0.001
LOS-ICU, Median (IQR)(d)	8.2 (4.9, 14.2)	8.2 (4.9, 14.6)	8.1 (4.7, 13.6)	0.136
Ventilation type, n (%)				< 0.001
Supplemental oxygen	1655 (43.5)	1413 (50.7)	242 (23.8)	
Invasive ventilation	2004 (52.7)	1263 (45.3)	741 (72.8)	
Tracheotomy	63 ( 1.7)	46 (1.7)	17 (1.7)	
High flow	82 ( 2.2)	64 (2.3)	18 (1.8)	
Ventilation time, Median (IQR)	3.0 (2.4, 4.5)	2.9 (2.4, 4.2)	3.4 (2.5, 5.4)	< 0.001
SpO2, Median (IQR)	97 (96, 99)	97 (96, 98)	98 (96, 99)	< 0.001

*GCS* Glasgow Coma Scale, *ICH* intracerebral hemorrhage, *ICU* intensive care unit, *IQR* interquartile range, *LOS* length of stay, *SAH* subarachnoid hemorrhage, *SOFA score* Sequential Organ Failure Assessment, *TBI* traumatic brain injury

**Fig. 2:**
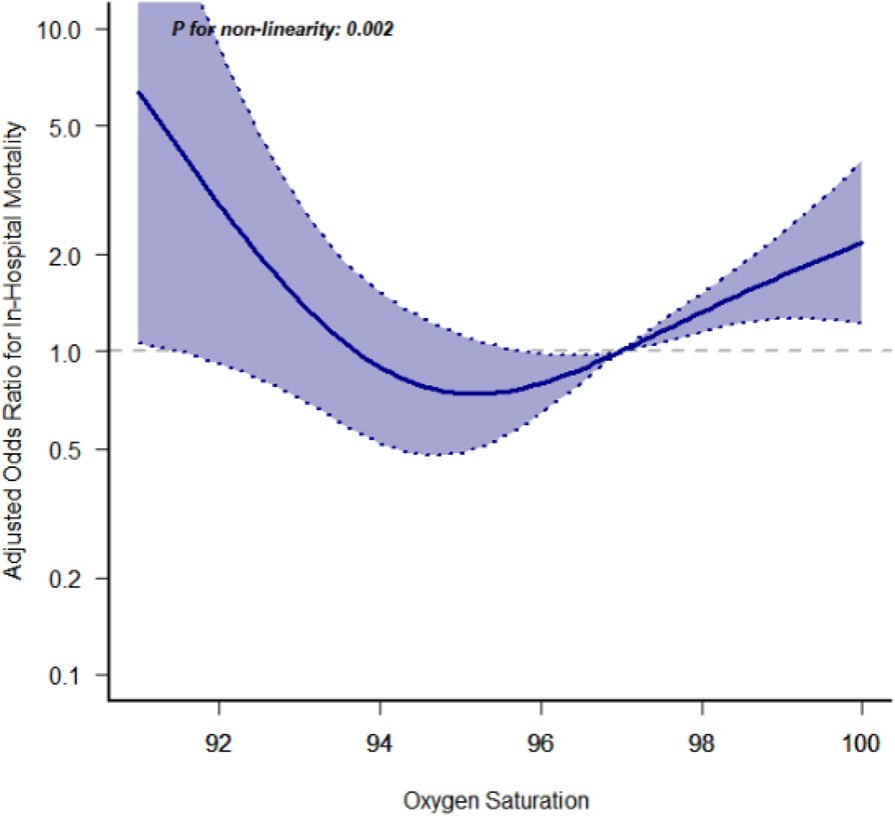
Association of the SpO2 and in-hospital mortality in all participants

Figure 2 shows the association between mortality and median SpO2. While hypoxemia is linked to a higher risk of death, hyperoxemia is also linked to a higher risk of death. Because of the impact of hypoxemia and hyperoxemia on mortality, a SpO2 range with a lower and higher limit has been established. While controlling covariates such as age, SOFA score, and GCS score on the first day of ICU admission, we utilized limited cubic splines to flexibly predict the link of SpO2 with mortality in patients with craniocerebral disorders. The relationship between SpO2 and in-hospital mortality was U-shaped for all subjects (*p* for nonlinearity < 0.001). According to limited cubic splines, the nadir for in-hospital mortality risk is 94%-96%. Less steep U-shaped curves are present as saturation levels rise. Even though we lack information on associated PaO2, we might assume that hyperoxia is less harmful than hypoxia. In Table 2, model 1 and model 2 shows that SpO2 in range of 94%-96% have a lower risk than other range of SpO2.

**Table 2:** Hazard ratio (95% CI) of in-hospital mortality according to ­SpO2 in all participants

SpO2	Number of patients	Numberof deaths	Mortality (%)	Model 1 HR (95%CI) *P* value		Model 2 HR (95%CI) *P* value	
< 92	10.0	6	60	0.91 (0.78 ~ 1.07)	0.267	0.05 (0 ~ Inf)	1.0
92–94	92.0	29	31.5	1.19 (0.47 ~ 2.97)	0.716	1.38 (0.42 ~ 4.5)	0.593
94–96	565.0	132	23.4	0.65 (0.46 ~ 0.93)	0.019	0.6 (0.41 ~ 0.88)	0.008
96–98	1441.0	328	22.8	0.96 (0.77 ~ 1.19)	0.693	0.94 (0.75 ~ 1.17)	0.58
≥ 98	1696.0	523	30.8	1.1 (0.98 ~ 1.22)	0.097	1.08 (0.97 ~ 1.21)	0.161

Model 1: unadjusted. Model 2: age, SOFA score, and base excess

*CI* confidence interval, *HR* hazard ratio, *SOFA score* Sequential Organ Failure Assessment

**Fig. 3:**
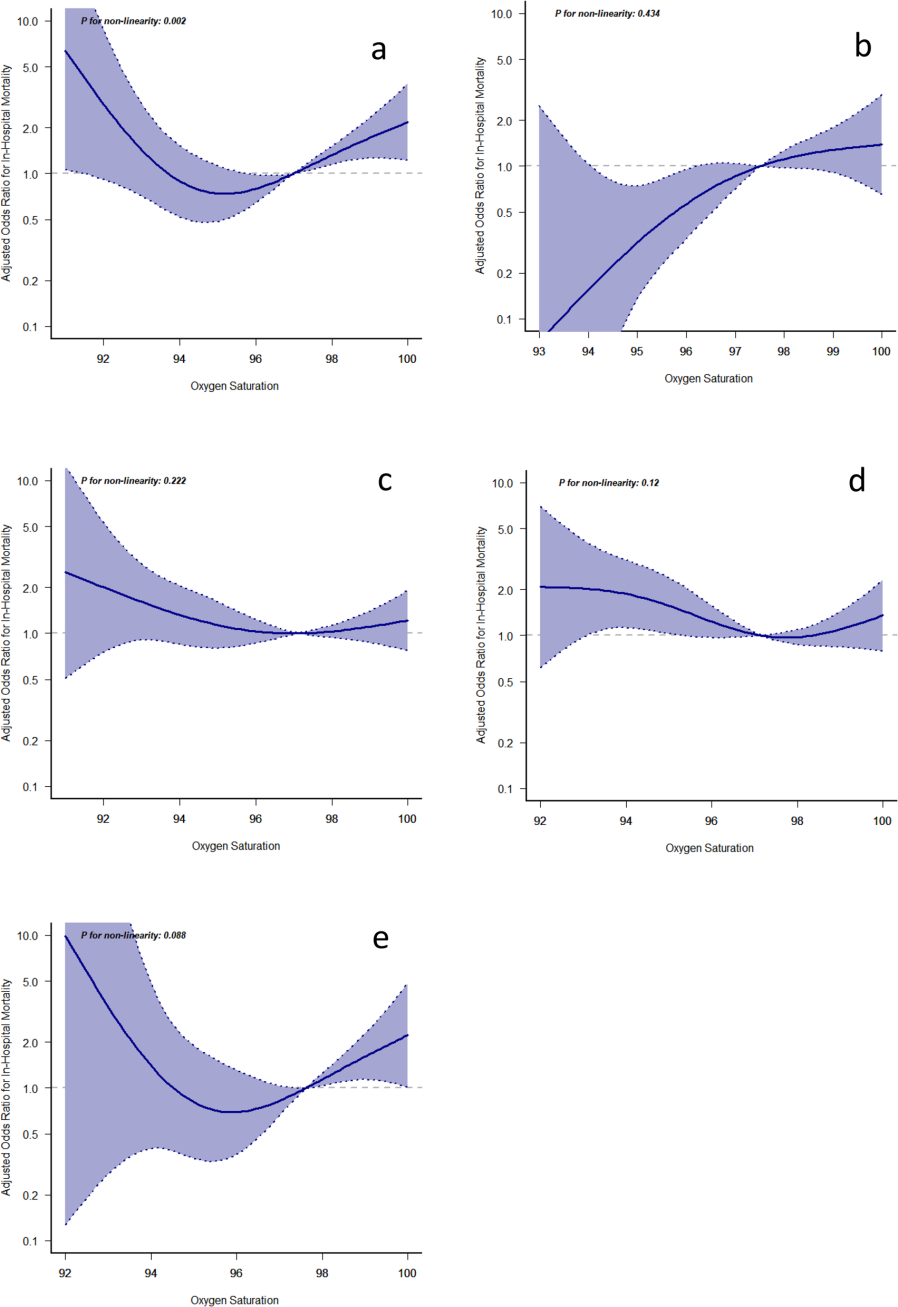
Association of the first 24-h ­SpO2 and in-hospital mortality in each subgroup. **a** Cerebral Infarction. **b** Intracerebral hemorrhage. **c** Metabolic/toxic encephalopathy. **d** Other Encephalopathy. **e** Subarachnoid hemorrhage

After that, we applied the same approach to investigate at each subgroup, and we discovered a U-shaped relationship between SpO2 and in-hospital mortality in patients with ICH, metabolic or toxic encephalopathy, SAH, cerebral infarction, and other encephalopathies (Fig. 3). Furthermore, we calculated the mortality risk to achieve a nadir at SpO2 in the 94–96% range, with negative and positive relationships below and above. The SpO2 of patients with traumatic brain injury and patients with SAH showed a U-shaped correlation with in-hospital mortality, and the in-hospital mortality was increased in the range of SpO2 below 94 and above 96. However, SpO2 in patients with ICH was not significantly associated with in-hospital mortality, Patients with metabolic/toxic encephalopathy, as well as other patients, showed lower in-hospital mortality in the SpO2 range of 96–98, with a U-shaped association. This is an intriguing finding: individuals with cerebral infarction and SAH have U-shaped curves with nadirs at 94–96% of saturation. In actuality, a severe ischemia burden for the brain characterizes both clinical disorders. The mortality data could be explained by ischemic tissue being particularly vulnerable to oxygen free radical damage. Maybe doctors should be very careful while titrating oxygen in these two categories of patients.

## Discussion

After controlling for other factors, our data from MIMIC-IV database show that SpO2 does have a U shape and is associated with in-hospital mortality in patients with craniocerebral diseases. In-hospital mortality was lowest when SpO2 was in the range of 94–96%. Different kinds of oxygen treatment had comparable effects. SpO2 levels that are too high or too low have a negative impact on patient survival. In a cohort of patients suffering from brain damage, our research offered additional evidence for the link between the SpO2 and in-hospital mortality. Given the scarcity of randomized control studies, this notion of an optimum SpO2 range will need to be tested further.

The correlation between SpO2 and PaO2 would be fair for a SpO2 range of 94–98%, with low chance of underestimating hypoxemia or hyperoxemia [13]. SpO2 is also more therapeutically useful since variations in SpO2 are used to alter inspired oxygen and ventilator settings rather than intermittent arterial blood gas measurements.

We used to think that the higher the oxygen saturation, the better the survival rate of patients, but on the contrary, studies have shown that the survival rate of patients with oxygen saturation in the range of 97–100% is lower than that of patients with oxygen saturation in the range of 94–98% [14]. Previous evidence suggests that oxygen saturation in hospitalized patients is responsible for multiple adverse outcomes, including increased length of stay and increased in-hospital mortality [15, 16]. Patients needing oxygen treatment may benefit from a SpO2 target of 94 to 98% [17]. This is exactly in line with the results of our research, which showed that higher oxygen saturation is not necessarily associated with better patient survival.

As we all know, a lack of oxygen causes a variety of problems in the human body's different systems. Hypoxia has various effects on the body, and different degrees of hypoxia have different consequences for the body [18]. For mild hypoxia, the lungs will experience deeper and faster breathing, and over time there will be fatigue of the respiratory muscles. Chronic mild hypoxia promotes brain vascular remodeling, although it also causes transitory vascular disruption [19]. Many studies have shown that prolonged hypoxia may have a pathogenic role in disrupting the blood–brain barrier and resulting in neurological impairment [20]. The impact on the cardiac circulatory system of human is mainly manifested as increased heart rate, increased myocardial contractility, decreased diastolic function, and the appearance of arrhythmia at the initial stage [21]. Normobaric hypoxia induces a fast drop in high-energy phosphate metabolism in the left ventricle of the human heart, which may result in a deterioration in diastolic function [22]. TBI often causes considerable damage to the brain's vasculature, resulting in cerebral hypoperfusion, ischemia, hypoxia, and other complications. However after moderate and severe TBI, hypoxia is most likely the main cause for vasculogenesis [23].

Hypoxia affects the central nervous system more than the rest of the body. The abnormal metabolism of brain tissue cells caused by hypoxia will further lead to the occurrence of brain edema and damage the blood–brain barrier [24].

The toxicity of O2 is frequently underestimated. Oxygen is seen as a necessity for human survival because mitochondria in the human body consume oxygen to produce the direct source of energy —ATP [25]. For oxygen saturation, higher is not better. From a micro perspective, reactive oxygen species (ROS), which are produced as by-products of aerobic metabolism or by specialized enzymes, can damage critical biological components such as proteins, lipids, and DNA(deoxyribonucleic acid) [26]. ROS is a double-edged sword[27, 28]. High blood oxygenation may cause vasoconstriction of major arterial beds, such as the cerebral or coronary, in terms of the big picture [6]. Because of increased oxidative stress and inflammation, excessive oxygen supplementation may have negative pulmonary and systemic consequences [29]. Hyperoxia has been found to produce central nervous system harm at a distance [30]. There is no definitive study available at this time that can explain the process by which hyperoxia causes harm to brain tissue. When the body's natural defensive mechanisms are overcome by prolonged exposure to severe hyperoxia, tissue injury and subsequent neuronal impairment are the inevitable outcomes [31]. Although there is no direct evidence that increased oxygen saturation leads to an increased chance of seizures, however studies have shown that patients with craniocerebral disease who are treated with hyperbaric oxygen in a hyperbaric oxygen chamber will lead to epileptic seizures due to the increase in oxygen partial pressure and exposure time [32]. When the patient is continuously hyperoxygenated, the risk of oxygen toxicity in the patient will be greatly increased. Central nervous system oxygen toxicity (CNS-OT) manifests as a variety of non-convulsive symptoms, many of which seem to originate in the brainstem and involve cranial nerve nuclei, autonomic and cardiorespiratory centers, before spreading to higher cortical regions and culminating in generalized tonic–clonic seizures [33]. However, since normobaric exposures do not result in CNS toxicity, we do not consider that excessive oxygen saturation will lead to CNS-OT [34]. Many studies show that cerebral hyperoxic vasoconstriction occurs for high PaO2 [35–37] and can also be used as a reference for SpO2.

The onset characteristics of patients with craniocerebral diseases are rapid onset and severe disease [38]. Failure to timely and correct treatment will endanger the patient's life. SpO2 can reflect the degree of oxygen saturation of hemoglobin in the patient's blood. Cerebral oxygen saturation is usually measured by jugular venous oxygen saturation (SvjO2), brain tissue oxygen tension (PtiO2), near infrared spectroscopy (NIRS), etc [39]. The current research has certain limitations. Looking forward to follow-up studies to determine the most adaptive target for cerebral oxygen saturation maintenance. At the same time, all our research data come from the MIMIC IV database, and all the patients are from the same medical center, so the conclusions drawn may not be applicable to patients in other regions. Moreover, the conclusions drawn from this retrospective analysis need to be further verified by more follow-up prospective experiments. There are still some data that have a certain impact on the patient's oxygen saturation, but due to the incomplete data, we will continue to study in the follow-up work.

## Conclusions

For patients with encephalopathy, too low or too high blood oxygen saturation will have adverse effects on the patient. When the oxygen saturation is maintained at 94–96%, TBI and SAH patients’ mortality rate will be minimized. However, more experiments are still needed to verify this conclusion.

## Data Availability

The datasets generated and/or analyzed during the current study are available in the MIMIC-IV (https://mimic-iv.mit.edu) databases.
